# Image encryption scheme based on improved four-dimensional chaotic system and evolutionary operators

**DOI:** 10.1038/s41598-024-57756-x

**Published:** 2024-03-25

**Authors:** Ying Niu, Hangyu Zhou, Xuncai Zhang

**Affiliations:** 1 School of Architecture Environment Engineering, Zhengzhou University of Light Industry, Zhengzhou, 450002 China; 2 School of Electrical and Information Engineering, Zhengzhou University of Light Industry, Zhengzhou, 450002 China

**Keywords:** Computational biology and bioinformatics, Mathematics and computing

## Abstract

To enhance the security of image data transmission, and address the weaknesses of existing image encryption schemes based on chaotic systems, particularly concerning resistance to differential attacks and the unstable performance of chaotic systems, this article introduces an improved four-dimensional chaotic system and integrates evolutionary operators to propose an image encryption scheme. Firstly, a method for generating pseudo-random sequences associated with the plaintext is designed. The change rate of the ciphertext pixel value exceeds 0.9967 after a slight modification of the plaintext pixel value, significantly improving the plaintext sensitivity and the scheme's ability to resist selected plaintext attacks. Secondly, an individual rearrangement operation is introduced to achieve bit-level scrambling, and pixel-level scrambling is achieved by selection strategy. Subsequently, crossover and mutation operations are incorporated into image encryption. To reflect the randomness of the pairing, we adopt the pseudo-random sequence generated by the chaotic system to control the crossover and mutation operators, and a diffusion operation is performed on selected pixel pairs. Finally, ciphertext feedback is applied. Experimental results and performance analysis demonstrate that the proposed scheme not only enhances the security of encrypted images but also effectively resists noise and cropping attacks. This method effectively meets the high-security requirements of images in network transmission and provides new ideas for further research in the field of image encryption.

## Introduction

Images, as an essential form of multimedia data, encompass a wide range of sensitive information, including personal privacy, business secrets, and medical images. With the continuous development of communication technology and the widespread use of information transmission, ensuring the security and confidentiality of image information has become particularly urgent. Image encryption, as a crucial technology in the field of information security, is essential to protect the safe transmission of such vital information. Due to the characteristics of images, such as massive data volume, strong correlation, high redundancy, and distinctive recognition features, traditional text encryption methods like AES and DES prove to be slow and ineffective in encrypting and decrypting images. These methods can no longer meet the encryption needs of large-capacity image data^1^. Consequently, researchers have begun exploring new methods for image encryption. Common approaches include image encryption schemes based on techniques such as wavelet transform encryption^2–4^, invertible transform^5–7^, deep learning^8–10^, quantum encryption^11–13^, and others. Chaotic systems have garnered significant attention in the field of information security due to their nonlinear dynamics and high sensitivity to initial conditions. In image encryption schemes, the integration of chaotic systems enhances unpredictability, thereby improving image security by introducing a certain degree of randomness. For instance, Wang^14^ proposed an image encryption scheme based on logistic map. This approach first utilizes wavelet transform to focus on the key information features of the image in the low-frequency part, subsequently encrypting the low-frequency information through the random sequence generated by logistic map. Mondal^15^ proposed an image encryption method based on two-dimensional Tent map, combined with meta-cellular automata. The random sequence generated by the Tent map serves as a cipher stream to control the state of each neighborhood cell and transforms each pixel value, achieving the encryption of the plaintext image. Although low-dimensional chaotic systems offer advantages in terms of simplicity, ease of implementation, and understanding, they have limitations in generating random sequences. These limitations may result in relatively low randomness and susceptibility to statistical analysis attacks.

To ensure the security of encrypted images and prevent malicious theft, some researchers have begun utilizing high-dimensional chaotic systems to design image encryption methods, addressing the limitations of low-dimensional chaotic systems. For example, Ma^16^ employed the three-dimensional Chen chaotic system and the Fisher-Yates permutation scheme to encrypt color images. In this approach, the sequence generated by the three-dimensional chaotic system serves as the cipher stream for the Fisher–Yates permutation scheme, disrupting the pixel positions. Subsequently, a new set of initial values is used to generate a new sequence through the chaotic system, ultimately operating with the plaintext pixel to alter its value. Three-dimensional chaotic systems provide higher security compared to their one-dimensional and two-dimensional systems, exhibiting more complex dynamical behavior, that enhances the system's unpredictability against potential attackers. Three-dimensional chaotic systems offer relatively robust protection with somewhat controllable computational complexity, making them suitable for moderately complex image encryption applications. However, although relatively secure, three-dimensional chaotic systems have a reasonably limited keyspace, which may need improvement in highly security-demanding scenarios. To overcome this deficiency, Zhao^17^ proposed a new image encryption method using a four-dimensional chaotic system combined with DNA coding. Experimental analyses demonstrate that the method not only achieves a substantial keyspace but also enhances security performance. Four-dimensional chaotic systems provide a larger keyspace than three-dimensional systems, heightening the difficulty of attacks and improving encryption security. As dimensionality increases, the dynamical behavior becomes more intricate, further strengthening the encryption. Four-dimensional chaotic systems are better suited for scenarios with higher security requirements than three-dimensional systems. They can provide richer dynamics, increasing the difficulty for attackers to predict the system's state. Some scholars have explored five or higher-dimensional chaotic systems^18–20^, where computational complexity grows exponentially with dimensionality. This may lead to a significant reduction in the real-time performance of image encryption in resource-constrained environments, such as embedded systems or mobile devices. Parameter tuning and optimization for high-dimensional chaotic systems are relatively more complex. Finding the right combination of parameters to ensure system stability and good cryptographic performance may require more time and computational resources. Additionally, higher-dimensional chaotic systems may be more demanding on hardware resources, which may limit applications on some resource-constrained devices. In practical applications of high-dimensional chaotic systems, these drawbacks and challenges should be comprehensively considered, balancing encryption security, computational efficiency, and hardware resource requirements.

As researchers delve deeper, they discover that single or structurally simple chaotic maps may have the potential to be less accurate and less secure. Therefore, image encryption methods are becoming increasingly diversified, typically employing not just one method but a combination of methods. Zhang^21^ utilized Latin square and S-box to implement pixel substitution and replacement, respectively, aiming to enhance the resistance of the encryption system against attacks. Wang^22^ employed a combination of dynamic parity row check and Z-transform to completely disrupt pixel positions. Additionally, the chunking method was used to diffuse different chunks of the ciphertext, thereby improving the robustness of the encryption system. Zhu^23^ applied chunk scrambling and an optimized artificial fish swarm scheme to double scramble pixel positions. Furthermore, the DNA coding technique was employed to diffuse each pixel value, enhancing overall security. Based on this, this article extends the traditional three-dimensional chaotic system into a new four-dimensional chaotic system by introducing new state variables based on the three-dimensional Lorenz chaotic system. Simultaneously, incorporating evolutionary operators and employing image encryption with the assistance of evolutionary operators such as selection, recombination, and mutation further enhances the effectiveness of image encryption. This fusion approach introduces a novel idea and method for research in the field of image encryption. To strengthen the cryptosystem and provide higher security, this work fully utilizes the properties of pseudo-randomness and the traversal of evolutionary operators and chaos theory. This comprehensive approach addresses the security threats and inefficiencies encountered in image encryption.

The main contributions of this article are as follows:

1. Proposing a new four-dimensional chaotic system by introducing new state variables and increasing the dimensionality of the system. This expansion results in a larger state space, offering increased degrees of freedom. Consequently, the system exhibits more complex trajectories with richer dynamical behavior.
2. Analyzing the maximum Lyapunov exponent with one and two parameters, sensitivity, and NIST test of the new four-dimensional chaotic system. The chaotic system demonstrates a high Lyapunov exponent and sensitivity to the initial key. The generated chaotic sequences exhibit high complexity and randomness, thereby enhancing the security of image encryption schemes.
3. Applying evolutionary operators to image encryption, utilizing sequences generated by the four-dimensional chaotic system to execute selection, mutation, and recombination operations on pixels. The approach facilitates highly random pixel value changes. Notably, the recombination operation involves eight rules, increasing the difficulty of cracking and improving the overall security of the encryption scheme.

The remaining sections of this article are outlined as follows: "Theoretical foundations" section introduces the concepts of three-dimensional Lorenz chaotic system and evolutionary operators; "Hyperchaotic system" section provides a detailed presentation of the proposed four-dimensional chaotic system and analyses its key performance metrics; "Image encryption scheme" section describes the detailed steps and procedures of the encryption scheme; "Simulation experiment results" section presents the experimental results and multiple security analyses; and "Conclusions" section concludes the article.

## Theoretical foundations

### Chaotic system

**Figure 1:**
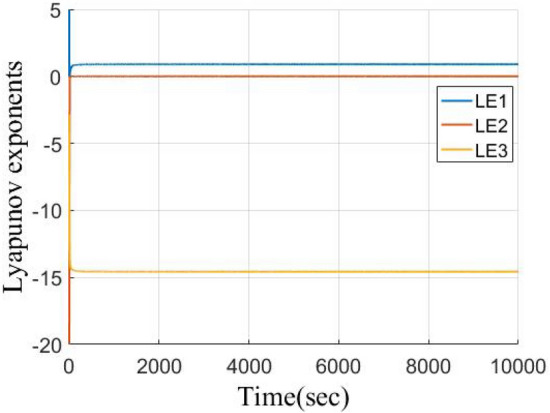
Lyapunov exponents of three-dimensional Lorenz chaotic system.

A chaotic system is a deterministic system characterized by seemingly random, irregular motions with attributes of uncertainty, irreducibility, and unpredictability. To strike a balance between the complexity and efficiency of chaotic systems, the Lorenz chaotic system, proposed by the meteorologist Edward Lorenz in his study of meteorology^24^, is a three-dimensional nonlinear dynamical system that describes a convective phenomenon in which air or liquid forms a complex vortex structure in a confined space. This system consists of three coupled differential equations with expressions, as shown in Eq. (1).

1 $$\begin{array}{*{20}c} {\left\{ {\begin{array}{*{20}c} {\dot{x} = \sigma \left( {y - x} \right) } \\ {\dot{y} = \gamma x - y - xz} \\ {\dot{z} = xy - bz } \\ \end{array} } \right.,} \\ \end{array}$$

where *x*, *y*, *z* are the state variables of the system, and *σ*, *γ*, *b* are the system's parameters. When the parameters are set to *σ* = 10, *γ* = 28, *b* = 8/3, with initial values of (1, 1, 1), the system exhibits classical chaotic phenomena, including extreme sensitivity to initial conditions and randomness. In a chaotic state, the system's trajectory displays complex and seemingly irregular motion, as depicted by its Lyapunov exponents in Fig. 1, which are (0.9021, − 0.0001, − 14.5686).

### Evolutionary strategy

**Figure 2:**
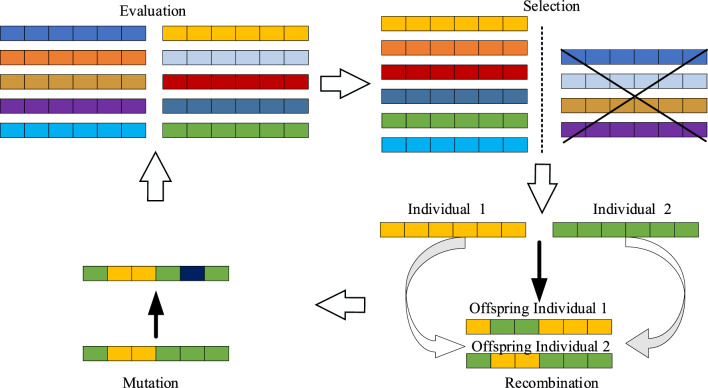
The main search loop of the evolutionary strategy.

Evolutionary strategy^25^ is an evolutionary computational method designed to tackle parameter optimization problems. The method draws inspiration from natural biological evolution and was first proposed by H.P. Schwefel of Germany in 1963. Evolutionary strategies find wide applicability across various optimization domains, including continuous, discrete, unconstrained, and constrained combinatorial search spaces, as well as hybrid search spaces. The core idea involves iteratively evolving a population of individuals containing candidate solutions through three key operators: selection, recombination, and mutation, to progressively refine solutions. The main search loop of the evolutionary strategy is illustrated in Fig. 2.

The evolutionary strategy comprises the following key steps:

1. *Population initialization* A group of individuals is randomly generated, with each representing a potential solution to the problem. The parameters of these individuals are chosen randomly.
2. *Fitness evaluation* Apply a problem-specific fitness function to each individual, to assess their performance in solving the problem. The fitness function quantifies the quality of individuals.
3. *Recombination* Involves the combination of some features or parameters of single or multiple individuals to generate new individuals.
4. *Mutation* Refers to the random or systematic perturbation of individual information to introduce new information and thus increase the diversity of the population.
5. *Selection* Selection is based on the individual's adaptation, and the better-adapted individuals are retained through recombination and mutation operations. This mimics the process of natural selection, wherein individuals with superior adaptations have a greater likelihood of survival and reproduction.
6. *Repeat iterations and algorithm termination* The above steps are repeated iterations, and the population evolves, with the better-adapted individuals being retained and continuously optimized to approach the optimal solution to the problem. The algorithm terminates when the maximum number of iterations, the adaptation threshold, or other specified conditions is reached; if the termination conditions are not reached, the algorithm returns to step 3.

Evolutionary strategies have strong local search ability and perform well in dealing with continuous optimization problems, enabling them to converge to the optimal solution quickly. Therefore, they are widely used in engineering optimization, machine learning, and computer simulation.

However, in the image encryption problem, although it is not a traditional optimization problem, the introduction of evolutionary operators aims to apply the selection, recombination, and mutation operations of biological evolution to image pixels. Encryption is achieved through pixel scrambling and diffusion via pixel selection, mutation of pixel values (expressed in binary form), and inter-pixel reorganization. Despite being applied to different domains, evolutionary operators demonstrate their flexibility and adaptability, successfully transitioning to the solution of the image encryption problem. Here, we define recombination operations in evolutionary operators for better application to image encryption, including:

1. *Individual rearrangement* This refers to the manipulation of the interior of a single individual to generate a new one. This can involve restructuring, parameter changes, etc.
2. *Crossover* It refers to selecting two or more individuals to generate new offspring by crossing over their parameters or structures. This emphasizes manipulation across individuals. Through crossover, evolutionary operators enable more flexible manipulation of pixels and produce a more comprehensive image encryption effect.

## Hyperchaotic system

### Proposed four-dimensional hyperchaotic system

**Figure 3:**
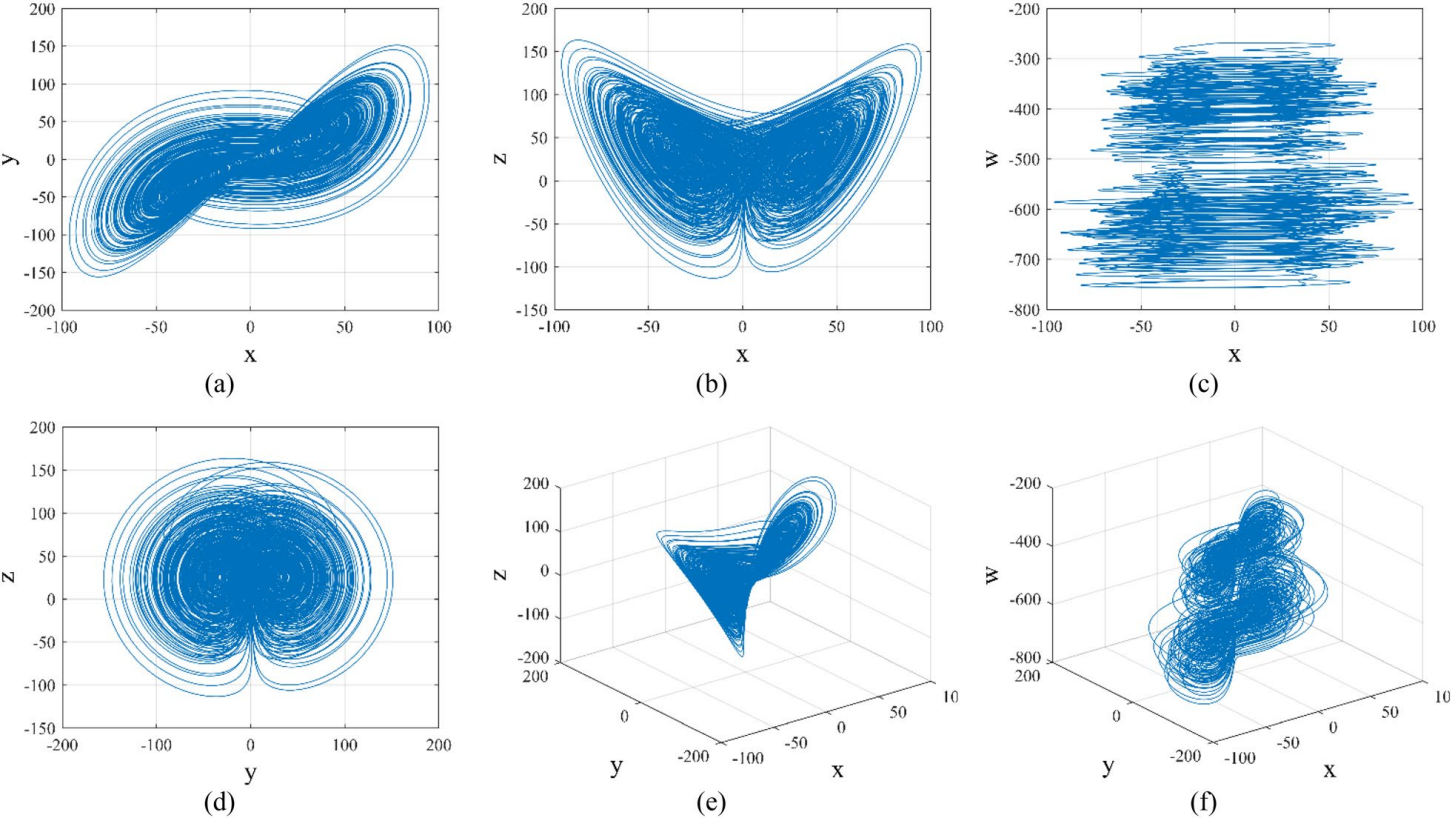
Phase diagram of hyperchaotic system: (**a**) *x*–*y* plane, (**b**) *x*–*z* plane; (**c**) *x*–*w* plane; (**d**) *y*–*z* plane; (**e**) *x*–*y*–*z* plane; (**f**) *y*–*z*–*w* plane.

As a nonlinear dynamical system, the three-dimensional Lorenz chaotic system has been widely utilized in image encryption due to its strong sensitivity to initial values and parameters, as well as its unpredictable trajectories. To further enhance the pseudo-randomness of the generated sequences, we introduce a new state variable, denoted as *w*, into the three-dimensional Lorenz system and couple it with the third dimension of the system to construct a four-dimensional hyperchaotic system. The state variable *w* interconnects the individual state variables and system parameters in a chaotic system to enhance the disorder and sensitivity of the chaotic system, whose expression is shown in Eq. (2).

2 $$\begin{array}{*{20}c} {\left\{ {\begin{array}{*{20}c} {\dot{x} = a\left( {y - x} \right) + z} \\ {\dot{y} = bx - cy - xz} \\ {\dot{z} = xy - dz + ew} \\ {\dot{w} = fx + y } \\ \end{array} } \right.,} \\ \end{array}$$

where *x*, *y*, *z*, and *w* represent the state variables of system, and *a*, *b*, *c*, *d*, *e* and *f* are the parameters controlling the chaotic behavior of the system. After parameter tuning, when the system parameters are set to *a* = 34, *b* = 28, *c* = 2.6, *d* = 4, *e* = 1.8, and *f* = 2.4, the system enters a hyperchaotic state with improved chaotic behavior. Given the initial values *x*_0_ = 0.1, *y*_0_ = 0.2, *z*_0_ = 0.2, and* w*_0_ = 0.2, the phase diagram of the hyperchaotic system is depicted in Fig. 3.

### Lyapunov exponent

**Figure 4:**
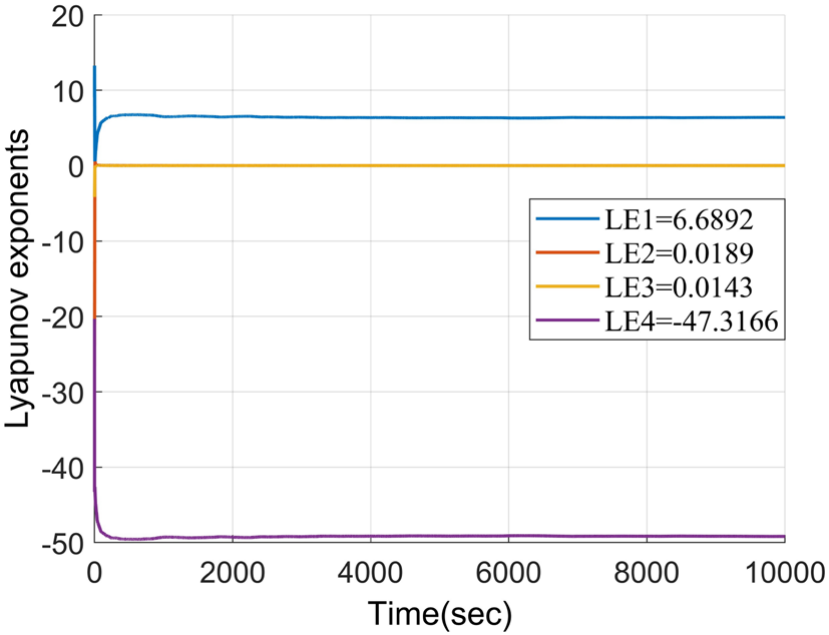
Lyapunov exponents of the improved hyperchaotic system.

The Lyapunov exponent quantitatively measures the dynamic properties of a system by assessing the rate of orbital mean dispersion. In a four-dimensional chaotic system, there are four Lyapunov exponents, with their sum being required to be less than zero, including at least two positive exponents^26^. Each parameter in the chaotic system has a distinct range, leading to different chaotic states and Lyapunov exponents. The Lyapunov exponents of the proposed four-dimensional chaotic system are depicted in Fig. 4, indicating LE1 = 6.6892, LE2 = 0.0189, LE3 = 0.0143, and LE4 = -47.3166. Since the sum is less than zero and there are three positive Lyapunov exponents, it can be inferred that the system enters a hyperchaotic state under these conditions.

The fractal dimension of the four-dimensional chaotic system, denoted as D_L_, can be computed using Eq. (3).

3 $$\begin{array}{*{20}c} {D_{L} = j + \frac{{\mathop \sum \nolimits_{i = 1}^{j} LE_{i} }}{{\left| {LE_{j + 1} } \right|}} = 3 + \frac{{LE_{1} + LE_{2} + LE_{3} }}{{\left| {LE_{4} } \right|}} = 3.1421,} \\ \end{array}$$

where *j* + 1 represents the dimension of the chaotic system. If the fractal dimension falls within the range of 3 < D_L_ < 4, it signifies a hyperchaotic system^27^. Upon calculation, the proposed system's fractal dimension satisfies 3 < D_L_ < 4, affirming its hyperchaotic nature.

**Figure 5:**
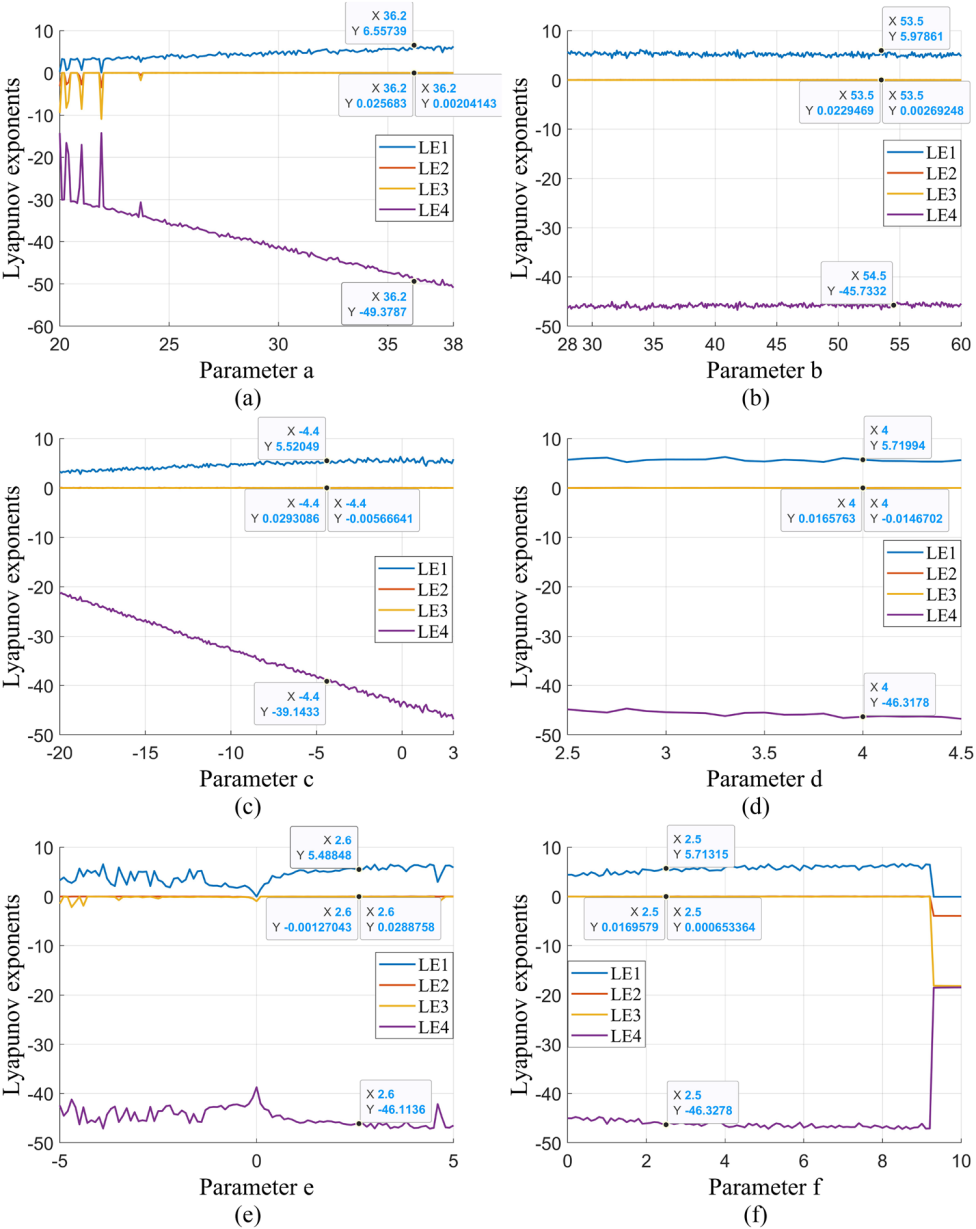
Single-parameter Lyapunov exponents: (**a**) parameter *a* ∈ [20, 38]; (**b**) parameter *b* ∈ [28, 60]; (**c**) parameter *c* ∈ [− 20, 3]; (**d**) parameter *d* ∈ [2.5, 4.5]; (**e**) parameter *e* ∈ [− 5, 5]; (**f**) parameter *f* ∈ [0, 10].

The single-parameter Lyapunov exponent diagram is employed to identify local maxima of a control parameter in a chaotic system, facilitating the visualization of the system's transition from cyclic to chaotic behavior. By leveraging these maxima, optimal parameter values can be selected to achieve an optimal chaotic state. Figure 5 illustrates the Lyapunov exponent for various parameters, showcasing the chaotic behavior induced by each parameter within a specified range. This analysis underscores the chaotic properties exhibited by the proposed four-dimensional chaotic system across the parameter spectrum, thereby enhancing the unpredictability of the generated pseudo-random sequences and expanding the keyspace.

The sequences generated by the three-dimensional Lorenz chaotic system often exhibit weak broadband characteristics, limited randomness, and monotonicity in localized regions^28^. To address these shortcomings, researchers have endeavored to enhance the three-dimensional Lorenz chaotic system for application in image encryption^8,29,30^. Table 1 compares the proposed four-dimensional chaotic system in this study with existing counterparts. Notably, the proposed system exhibits three positive Lyapunov exponents, whereas other existing systems feature only two. Additionally, the maximum Lyapunov exponent of the proposed system surpasses that of the others, indicating a more complex and nonlinear structure. Furthermore, the inclusion of multiple parameters in the proposed system results in a larger keyspace, bolstering image encryption security compared to other systems.

**Table 1:** Comparison of four-dimensional chaotic systems.

Chaotic systems	LE1	LE2	LE3	LE4	Number of parameters
Our	6.6892	0.0189	0.0143	− 47.3166	6
^29^	3.3488	0.1399	0	− 17.1252	6
^30^	0.2000	0.3000	0	− 7.5600	4
^8^	0.3300	0.1586	0	− 15.1752	4

### Maximum Lyapunov exponent for two-parameter

**Figure 6:**
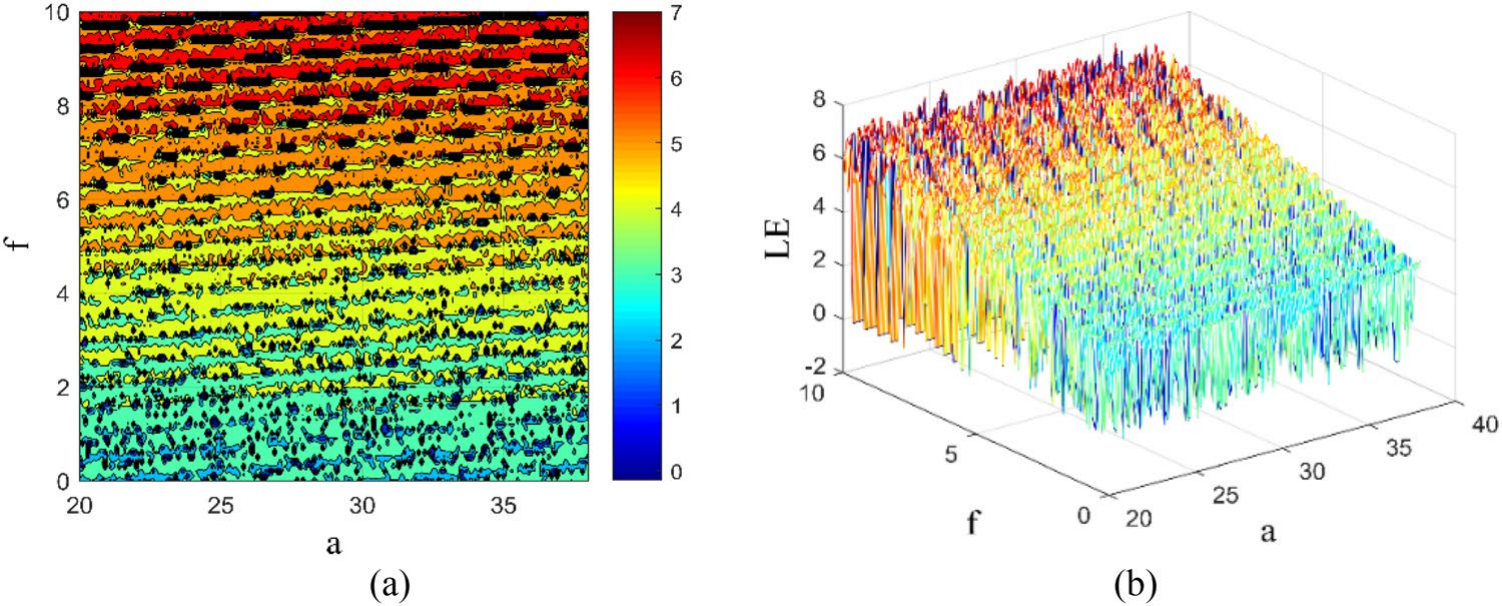
Two-parameter Lyapunov exponents: (**a**) maximum Lyapunov exponent diagram in (*a*–*f*) plane; (**b**) three-dimensional visualization diagram in (*a*–*f*) plane.

Chaotic systems are nonlinear dynamical systems characterized by intricate behavior and high sensitivity to system parameters. Even minor alterations in these parameters can significantly influence the system's trajectory. The two-parameter maximum Lyapunov exponent diagram offers insight into the dynamic behavior of chaotic systems. By setting initial values at (0.1, 0.2, 0.2, 0.2), the impact of parameters *a* ∈ [20, 37] and *f* ∈ [0, 10] on the system's behavior is investigated. Figure 6 illustrates the two-parameter maximum Lyapunov exponent diagram and its three-dimensional representation, with distinct colors denoting different Lyapunov exponents: darker shades (red) indicating larger exponents, and lighter shades (blue) indicating smaller ones.

### Sensitivity analysis

**Figure 7:**
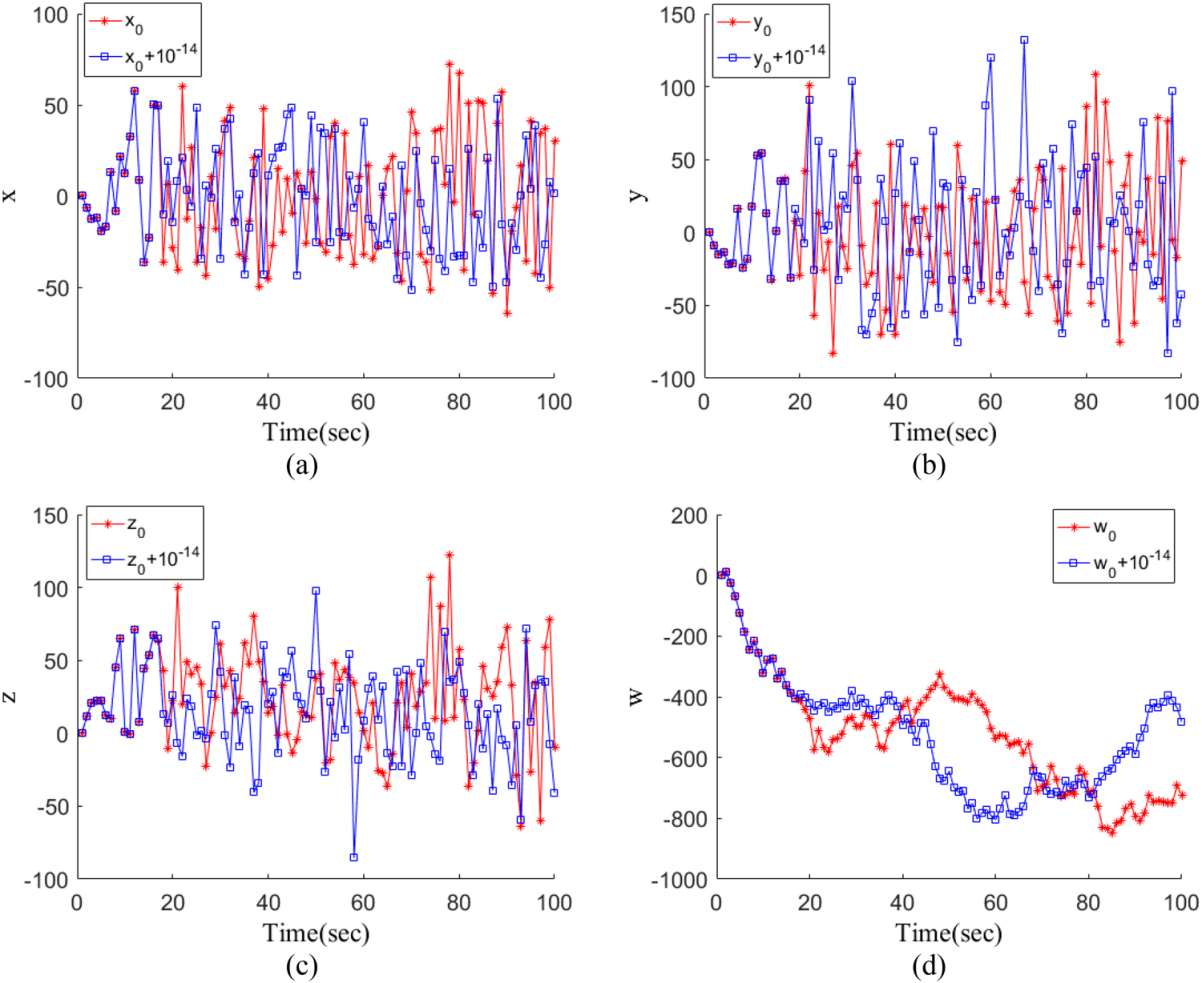
Initial value sensitivity test: (**a**) ${x}_{0}+{10}^{-14}$; (**b**) ${y}_{0}+{10}^{-14}$; (**c**) ${z}_{0}+{10}^{-14}$; (**d**) ${w}_{0}+{10}^{-14}$.

When varying initial values within a chaotic system, different sequences are generated, underscoring the system's sensitivity to these values. Minor adjustments to initial values lead to distinct chaotic sequences, highlighting the system's high sensitivity. In this section, slight modifications are made to the initial values, with four sets of tests conducted using *x*_0_ (0.1), *y*_0_ (0.2), *z*_0_ (0.2), *w*_0_ (0.2), and *x*_0_ + 10^–14^, *y*_0_ + 10^–14^, *z*_0_ + 10^–14^, *w*_0_ + 10^–14^. The outcomes of these tests are depicted in Fig. 7. Notably, even minute changes in individual initial values yield markedly different sequences, underscoring the pronounced sensitivity of the proposed four-dimensional chaotic system to initial conditions.

### Dissipative and equilibrium stability analysis

The total energy of the chaotic system diminishes over time, while the phase space contracts as the system evolves. This characteristic, known as the dissipative nature of a chaotic system^31^, is defined by Eqs. (4) and (5):

4 $$\begin{array}{*{20}c} {\nabla V = \frac{{\partial \dot{x}}}{\partial x} + \frac{{\partial \dot{y}}}{\partial y} + \frac{{\partial \dot{z}}}{\partial z} + \frac{{\partial \dot{w}}}{\partial w} < 0,} \\ \end{array}$$

5 $$\begin{array}{*{20}c} {\frac{dV}{{dt}} = e^{\nabla V} ,} \\ \end{array}$$

where *t* represents time, and *V* denotes the dissipation volume. By substituting the system parameters, *a* = 34, *b* = 28, *c* = 2.6, *d* = 4, *e* = 1.8, and *f* = 2.4 into Eq. (4), we obtain ∇*V* = − *a *− *c *− *d* = − 40.6, indicating that ∇*V* < 0. Therefore, at this juncture, the chaotic system is dissipative. As time progresses infinitely, the chaotic system's phase space eventually converges to a point, forming an attraction domain. Modifying one aspect of the chaotic system to 0, as depicted in Eq. (6):

6 $$\begin{array}{*{20}c} {\left\{ {\begin{array}{*{20}c} {a\left( {y - x} \right) + z = 0} \\ {bx - cy - xz = 0 } \\ {xy - dz + ew = 0 } \\ {fx + y = 0 } \\ \end{array} } \right..} \\ \end{array}$$

After computation, the chaotic system yields two equilibrium points: *E*_1_ = (0, 0, 0, 0) and *E*_2_ = (0.2962, − 0.7109, 34.2400, 76.2059). The chaotic system’s Jacobian matrix is shown in Eq. (7):

7 $$\begin{array}{*{20}c} {J = \left[ {\begin{array}{*{20}c} { - a} & a & 1 & 0 \\ {b - z} & { - c} & { - x} & 0 \\ y & x & { - d} & e \\ f & 1 & 0 & 0 \\ \end{array} } \right].} \\ \end{array}$$

With control parameters *a* = 34, *b* = 28, *c* = 2.6, *d* = 4, *e* = 1.8, and *f* = 2.4, the eigenvalues of each equilibrium point are computed using the Jacobian matrix. For *E*_1_, the four eigenvalues are λ_1_ = − 52.9183, λ_2_ = 16.3249, λ_3_ = -0.0179, λ_4_ = − 3.9887, while for *E*_2_, the corresponding eigenvalues are λ_1_ = − 24.1106, λ_2_ = − 12.4377, λ_3_ = − 4.1019, and λ_4_ = 0.0501.

According to the Routh-Hurwitz criterion^32^, the coefficients of the eigen equations must all be negative for equilibrium points to be stable. However, not all coefficients of the eigenvalues for both equilibrium points are negative, indicating that they are unstable.

### NIST test

The randomness of the sequence can be quantitatively assessed using the NIST randomization test^33^. In this experiment, the NIST test was conducted on four sequences generated by the four-dimensional chaotic system. Prior to the test, these sequences were converted into binary sequences. If *P* > 0.01, it indicates that the sequence is sufficiently random to pass the NIST test.

Table 2 displays the test results, indicating that the *P* values for all test items are highly significant, surpassing the significance level of 0.01. This verifies the randomness of the chaotic sequence, ensuring the encryption’s security.

**Table 2:** NIST random test results.

NIST-Nnme	*P* value				Result
	*x*	*y*	*z*	*w*	
Frequency	0.976060	0.213309	0.739918	0.082177	Pass
Block frequency	0.407091	0.082177	0.082177	0.671779	Pass
Cumulative sums	0.602458	0.804337	0.671779	0.534146	Pass
Runs	0.671779	0.213309	0.949602	0.213309	Pass
Longest run	0.066882	0.253551	0.739918	0.213309	Pass
Rank	0.213309	0.911413	0.407091	0.066882	Pass
FFT	0.862344	0.949602	0.100508	0.299251	Pass
Nonperiodic template	0.911413	0.862344	0.949602	0.949602	Pass
Overlapping template	0.739918	0.299251	0.299251	0.534146	Pass
Universal statistical	0.468595	0.534146	0.213309	0.468595	Pass
Approximate entropy	0.739918	0.122325	0.468595	0.862344	Pass
Random excursions	0.122325	0.964295	0.739918	0.350485	Pass
Random excursions variant	0.122325	0.834308	0.534146	0.213309	Pass
Serial	0.976060	0.299251	0.862344	0.739918	Pass
Linear complexity	0.602458	0.407091	0.534146	0.100508	Pass

## Image encryption scheme

**Figure 8:**
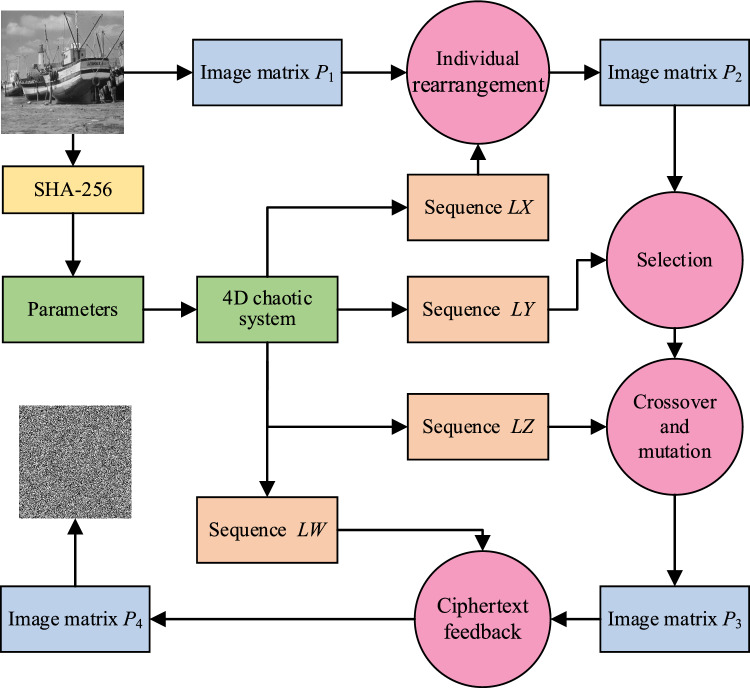
Flowchart of encryption scheme.

The generation of initial parameters for the chaotic system involves combining the provided external key with the plaintext image to generate pseudo-random sequences associated with the plaintext. Subsequently, the plaintext undergoes scrambling through individual rearrangement and selection operations, resulting in double scrambling at both the bit-level and pixel-level. Additionally, the image is subjected to crossover and mutation operations as part of the image diffusion process, aiming to obscure the original image. The inclusion of pseudo-random sequences in the ciphertext feedback further aids in the diffusion process. The detailed encryption flowchart is depicted in Fig. 8.

### Key generation

To ensure that different keys are used for different images, we associate the key with the plaintext and use the plaintext image pixel values and hash values to update the key. Using the SHA-256 scheme, a 256-bit binary hash value (named *K*) is obtained by performing encryption operations on the plaintext image. Using this operation to perform key updates in every encrypt round. *K* is divided into 32 groups of 32 bytes by byte, each represented as *K*_1_, *K*_2_, …, *K*_32_. According to Eqs. (8)–(10), the updated set of keys is computed to get the updated set of keys for the chaotic system and the initial values.

8 $$\left\{ {\begin{array}{*{20}l} {Q_{1} = \frac{4}{N}\mathop \sum \limits_{i = 1}^{\frac{N}{4}} q_{i} - \left[ {\frac{4}{N}\mathop \sum \limits_{i = 1}^{\frac{N}{4}} q_{i} } \right]} \hfill \\ {Q_{2} = \frac{4}{N}\mathop \sum \limits_{{i = \frac{N}{4} + 1}}^{\frac{N}{2}} q_{i} - \left[ {\frac{4}{N}\mathop \sum \limits_{{i = \frac{N}{4} + 1}}^{\frac{N}{2}} q_{i} } \right]} \hfill \\ {Q_{3} = \frac{4}{N}\mathop \sum \limits_{{i = \frac{N}{2} + 1}}^{\frac{3N}{4}} q_{i} - \left[ {\frac{4}{N}\mathop \sum \limits_{{i = \frac{N}{2} + 1}}^{\frac{3N}{4}} q_{i} } \right]} \hfill \\ {Q_{4} = \frac{4}{N}\mathop \sum \limits_{{i = \frac{3N}{4} + 1}}^{N} q_{i} - \left[ {\frac{4}{N}\mathop \sum \limits_{{i = \frac{3N}{4} + 1}}^{N} q_{i} } \right]} \hfill \\ \end{array} } \right.,$$

9 $$\left\{ {\begin{array}{*{20}l} {\alpha = \frac{{\left( {K_{1} \oplus K_{2} \oplus K_{3} \oplus K_{4} } \right) + K_{5} + K_{6} + K_{7} + K_{8} }}{256*5}} \hfill \\ {\beta = \frac{{\left( {K_{9} \oplus K_{10} \oplus K_{11} \oplus K_{12} } \right) + K_{13} + K_{14} + K_{15} + K_{16} }}{256*5}} \hfill \\ {\gamma = \frac{{\left( {K_{17} \oplus K_{18} \oplus K_{19} \oplus K_{20} } \right) + K_{21} + K_{22} + K_{23} + K_{24} }}{256*5}} \hfill \\ {\omega = \frac{{\left( {K_{25} \oplus K_{26} \oplus K_{27} \oplus K_{28} } \right) + K_{29} + K_{30} + K_{31} + K_{32} }}{256*5}} \hfill \\ \end{array} } \right.,$$

10 $$\left\{ {\begin{array}{*{20}l} {x_{0} = mod\left( {Q_{1} + Q_{2} - \alpha ,1} \right) + x^{\prime}_{0} } \hfill \\ {y_{0} = mod\left( {Q_{2} + Q_{3} - \beta ,1} \right) + y^{\prime}_{0} } \hfill \\ {z_{0} = mod\left( {Q_{3} + Q_{4} - \gamma ,1} \right) + z^{\prime}_{0} } \hfill \\ {\omega_{0} = mod\left( {Q_{4} + Q_{1} - \omega ,1} \right) + \omega^{\prime}_{0} } \hfill \\ \end{array} } \right.,$$

where [*x*] is a rounding function, *q*_*i*_ is determined by the average of the pixel values in the *i*th column of the plaintext image, *N* is the number of columns in the plaintext image, and *x*_0_', *y*_0_', *z*_0_', *ω*_0_' are given values.

### Selection operation

In evolutionary strategy, the selection operation refers to the process of selecting individuals from the population based on their adaptability. The goal of the selection operation is to maintain or improve the overall quality of the population over consecutive generations, guiding the evolutionary process towards an optimal solution. In image encryption methods, to enhance the confusion effect, we need to introduce more randomness. Therefore, we have improved the selection operation to better adapt to the requirements of image encryption. In this article, we utilize the pseudo-randomness of chaotic sequences to achieve individual selection, aiming to achieve better pixel scrambling.

Given an image *P* of size *M* × *N*, for traversing all the pixels, the selection in this article involves random traversal, differing from the selection in the optimization scheme. The randomness of selection is achieved here with the help of chaotic sequences due to their easy generation, strong sensitivity to initial conditions, and complete reproducibility.

**Figure 9:**
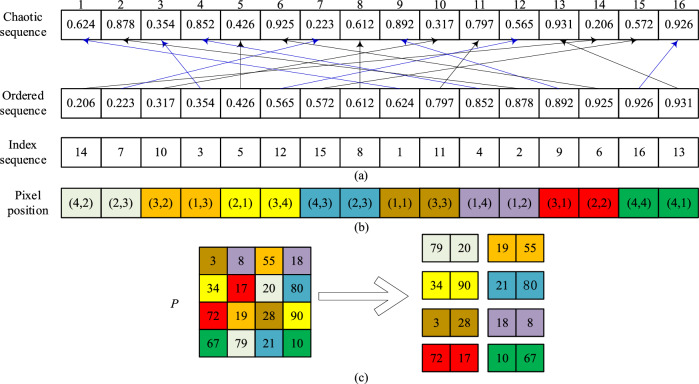
Schematic diagram of selection strategy: (**a**) the chaotic sequence, the ascending sequence, and the index sequence; (**b**) shows the pixel position pair representation corresponding to the 4 × 4 matrix; (**c**) the 4 × 4 pixel matrix and its pixel pair selection schematic.

To achieve this, rearrange the generated pseudo-random sequence in ascending order to obtain an ordered sequence. Determine the location of each element in the ordered sequence and its position in the original sequence. Then, form a new sequence from these position sequences in order, i.e., the position index sequence *Index* = {*Index*_1_, *Index*_2_, *Index*_3_, …* Index*_*M*×*N*_}. Based on the values of two adjacent pixels in the position index sequence, select the corresponding pixel positions in the image *P* according to Eq. (11) to obtain the pixel pairs *Pixel*_*A*_ and *Pixel*_*B*_. Figure 9 shows a schematic diagram of the selection strategy, where Fig. 9a shows the chaotic sequence, the ascending sequence, and the index sequence of length 4 × 4, Fig. 9b shows the pixel position pair representation corresponding to the 4 × 4 matrix, and Fig. 9c shows the 4 × 4 pixel matrix and its pixel pair selection schematic, where *P* is the plaintext image matrix. The selection formula (11) is described as follows:

11 $$\begin{array}{*{20}c} {\left\{ {\begin{array}{*{20}c} {Pixel_{A} = P\left( {floor\left( {(Index_{i} - 1)/N} \right) + 1,mod\left( {Index_{i} - 1,N} \right) + 1} \right) } \\ {Pixel_{B} = P\left( {floor\left( {(Index_{i + 1} - 1)/N} \right) + 1,mod\left( {Index_{i + 1} - 1,N} \right) + 1} \right)} \\ \end{array} } \right.,} \\ \end{array}$$

where *P*(·) is the plaintext pixel, *Pixel*_*A,*_ and *Pixel*_*B*_ are the selected pixel pairs, *mod*(·) is the modulo operation, *floor*(·) is the rounding operation, *i* is odd, and *i* < *M* × *N*.

### Crossover and mutation operation

In this article, crossover and mutation are utilized to alter the bit sequence, enhancing the robustness and security of encryption. Given two parents and a random number, two offspring individuals are obtained after crossover. Here, the individuals are the pixels in the image, each represented by 256 grey levels with pixel values in an 8-bit binary. Following the previous selection strategy, for the selected pixel pairs noted as *Pixel*_*A*_ = *a*_8_*a*_7_*a*_6_*a*_5_*a*_4_*a*_3_*a*_2_*a*_1_ and *Pixel*_*B*_ = *b*_8_*b*_7_*b*_6_*b*_5_*b*_4_*b*_3_*b*_2_*b*_1_, the pixel pairs producing the children are denoted as $\it{Pixel}^{ \prime}_{A}$ = $a^{\prime}_{8} a^{\prime}_{7} a^{\prime}_{6} a^{\prime}_{5} a^{\prime}_{4} a^{\prime}_{3} a^{\prime}_{2} a^{\prime}_{1}$ and $\it{Pixel}^{ \prime}_{B}$ = $b^{\prime}_{8} b^{\prime}_{7} b^{\prime}_{6} b^{\prime}_{5} b^{\prime}_{4} b^{\prime}_{3} b^{\prime}_{2} b^{\prime}_{1}$. The random number is noted as *C* = *c*_8_*c*_7_*c*_6_*c*_5_*c*_4_*c*_3_*c*_2_*c*_1_.

1. *Crossover* According to the random number *c*, if *c*_*i*_ = 0, pixel $\it{Pixel}^{ \prime}_{A}$ inherits the value of the corresponding binary bit of *Pixel*_*B*_, and pixel ${\it{Pixel}}^{ \prime}_{B}$ inherits the value of the corresponding binary bit of *Pixel*_*A*_; if *c*_*i*_ = 1, pixel $\it{Pixel}^{ \prime}_{A}$ inherits the value of the corresponding bit of *Pixel*_*A*_, and pixel $\it{Pixel}^{ \prime}_{B}$ inherits the value of the corresponding bit of Pixel_*B*_.
2. *Mutation* Non-uniform mutation is used, for the pixel to be varied, according to the random number *c*, if *c*_*i*_ = 0, the bit in which the new pixel is located inherits the value of the original pixel in the bit in which it is located; if *c*_*i*_ = 1, the corresponding bit of the pixel is varied: 0 becomes 1, or 1 becomes 0. Thus, two new individuals $\it{Pixel}^{ \prime}_{A}$ and $\it{Pixel}^{ \prime}_{B}$ are obtained.

**Figure 10:**
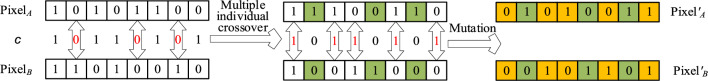
Schematic diagram of crossover and mutation.

The process of crossover and mutation is schematically shown in Fig. 10. For the selected pixel pairs, the same random number is used for crossover and mutation in this article, these operations can be performed simultaneously. Crossover and mutation can be described by Eqs. (12) and (13):

12 $$\begin{array}{*{20}c} {a^{\prime}_{i} = \left\{ {\begin{array}{*{20}c} {b_{i} \; if \quad c_{i} = 0} \\ {\widetilde{{a_{i} }} \; if\quad c_{i} = 1} \\ \end{array} } \right.,} \\ \end{array}$$

13 $$\begin{array}{*{20}c} {b^{\prime}_{i} = \left\{ {\begin{array}{*{20}c} {a_{i} \; if \quad c_{i} = 0} \\ {\widetilde{{b_{i} }}\; if\quad c_{i} = 1} \\ \end{array} } \right.,} \\ \end{array}$$

where *a*_*i*_ and *b*_*i*_ are the *i*th bit of the original pixel pairs, $\tilde{a_{i}}$ and $\tilde{b_{i}}$ are the results of the *a*_*i*_ and *b*_*i*_ non-operations, respectively, $a^{\prime}_{i}$ and $b^{\prime}_{i}$ are the *i*th bit of the new pixel pairs after crossover and mutation, and *i* is the number of bits in the binary sequence.

### Individual rearrangement operation

**Figure 11:**
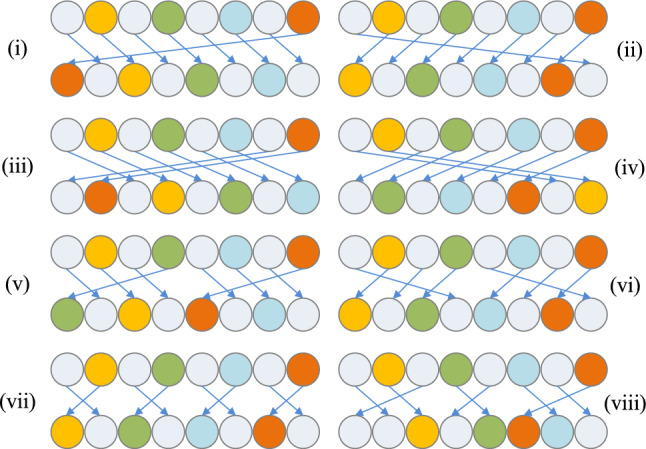
Individual rearrangement rules.

With the idea of rearrangement, the grey value of the pixel to be encrypted is converted into an 8-bit binary sequence. The binary sequence of the pixel undergoes individual rearrangement to achieve bit-level scrambling. Here, eight individual rearrangement rules are defined, as shown in Fig. 11. For a given pixel, one individual rearrangement rule is randomly selected to scramble its binary sequence, affecting the change in pixel value. The random selection is achieved using pseudo-random sequences generated by the chaotic system.

### Ciphertext feedback

Ciphertext feedback is an operation designed to enhance the interaction between pixels and alter pixel values. It allows small changes in plaintext to diffuse throughout the entire ciphertext, boosting the scheme's resistance to differential and statistical attacks. The process efficiently propagates small changes in the plaintext image to the entire ciphertext image by modifying the pixel value based on the previous pixel value along with the generated pseudo-sequence value. Given a pseudo-random sequence *D* = {*d*_1_, *d*_2_, *d*_3_,… *d*_*M*×*N*_} of length *M* × *N*, where the element values in *D* range from 0 to 255, the image matrix is converted into a one-dimensional sequence *S* = {*s*_1_, *s*_2_, *s*_3_,… *s*_*M*×*N*_} of length *M* × *N* in row-first order. The feedback image sequence is denoted as *C* = {*c*_1_, *c*_2_, *c*_3_,… *c*_*M*×*N*_}, and the feedback process is shown in Eq. (14):

14 $$\begin{array}{*{20}c} {c_{i} = s_{i} \oplus c_{i - 1} \oplus d_{i} ,} \\ \end{array}$$

where the initial element *c*_0_ = 127 and *i* = 1, 2, …, *M* × *N*.

### Complete the encryption process

The proposed image encryption scheme in this article comprises two parts: the evolutionary operators and the ciphertext feedback. The evolutionary operators involve selection, crossover, and mutation, performing double scrambling and diffusion at the bit and pixel levels. This encryption scheme is designed to encrypt images of any size. For any *M* × *N* image, if *M* × *N* is even, no processing is required; if *M* × *N* is odd, supplementary processing is done with '0'. The detailed encryption process is as follows:

Input a grey level image *P* of size *M* × *N*, where *M* and *N* are the number of rows and columns of the image, with initial values of the parameters *x*_0_', *y*_0_', *z*_0_' and *w*_0_'; Output a ciphertext image *E*.

- *Step 1* Convert the grey level image *P* to be encrypted into an image matrix of size *M* × *N*, denoted as matrix *P*_1_.
- *Step 2* Use the hash function to calculate the hash value *K* of matrix *P*_1_. Obtain the initial parameter values *x*_0_, *y*_0_, *z*_0_, and *w*_0_ of the chaotic system using Eqs. (8)–(10).
- *Step 3* The four-dimensional chaotic system is iterated *M* × *N* + 1000 times, and the values of the first 1000 iterations are discarded to eliminate transient effects, thereby obtaining four pseudo-random sequences *LX*, *LY*, *LZ*, and *LW* of length *M* × *N*.
- *Step 4* Process the pseudo-random sequence *LX* according to Eq. (15), reshaping it into a matrix form for the selection of individual recombination rules. After the individual recombination operation in "Individual rearrangement operation" section, recombine and scramble each pixel in matrix *P*_1_ to obtain the recombination matrix *P*_2_.
  15 $$\begin{array}{*{20}c} {lx_{i}^{\prime} = mod\left( {floor\left( {10^{14} \times lx_{i} } \right),8} \right) + 1.} \\ \end{array}$$
- *Step 5* Sort the pseudo-random sequence *LY* in ascending order to obtain a new sequence *LY*'. Find the positions of each element in the original sequence *LY* within the new sequence *LY*', and arrange these position numbers into a new sequence to obtain the index sequence ‘*Index*’. According to the index sequence ‘*Index*’, select the pixel pairs sequentially from the matrix *P*_2_ using Eq. (11).
- *Step 6* Process the pseudo-random sequence *LZ* according to Eq. (16) to obtain the random sequence *LZ*' and ensure the value of each element in the random sequence *LZ*' is between 0 and 255. According to "Crossover and mutation operation" section, select the elements from the odd positions of the random sequence *LZ*' as the random numbers used in the crossover and mutation operations. Based on the selected random numbers, the pixels selected in Step 5 are sequentially subjected to crossover and mutation operations to obtain the matrix *P*_3_.
  16 $$\begin{array}{*{20}c} {lz_{i}^{\prime} = mod\left( {floor\left( {10^{14} \times lz_{i} } \right),256} \right).} \\ \end{array}$$
- *Step 7* Process the pseudo-random sequence *LW* according to Eq. (17). Convert matrix *P*_3_ into a one-dimensional sequence, and according to the ciphertext feedback operation described in "Ciphertext feedback" section, perform ciphertext feedback for each pixel. Recover the result into matrix form to obtain matrix *P*_4_, i.e., the ciphertext image *E*.
  17 $$\begin{array}{*{20}c} {lw_{i}^{\prime} = mod\left( {floor\left( {10^{14} \times lw_{i} } \right),256} \right).} \\ \end{array}$$

The decryption scheme is the inverse process of the above scheme and is not elaborated here. Additionally, this scheme is equally applicable to encrypting color images by simply decomposing pixels that are only images into RGB channels.

## Simulation experiment results

**Figure 12:**
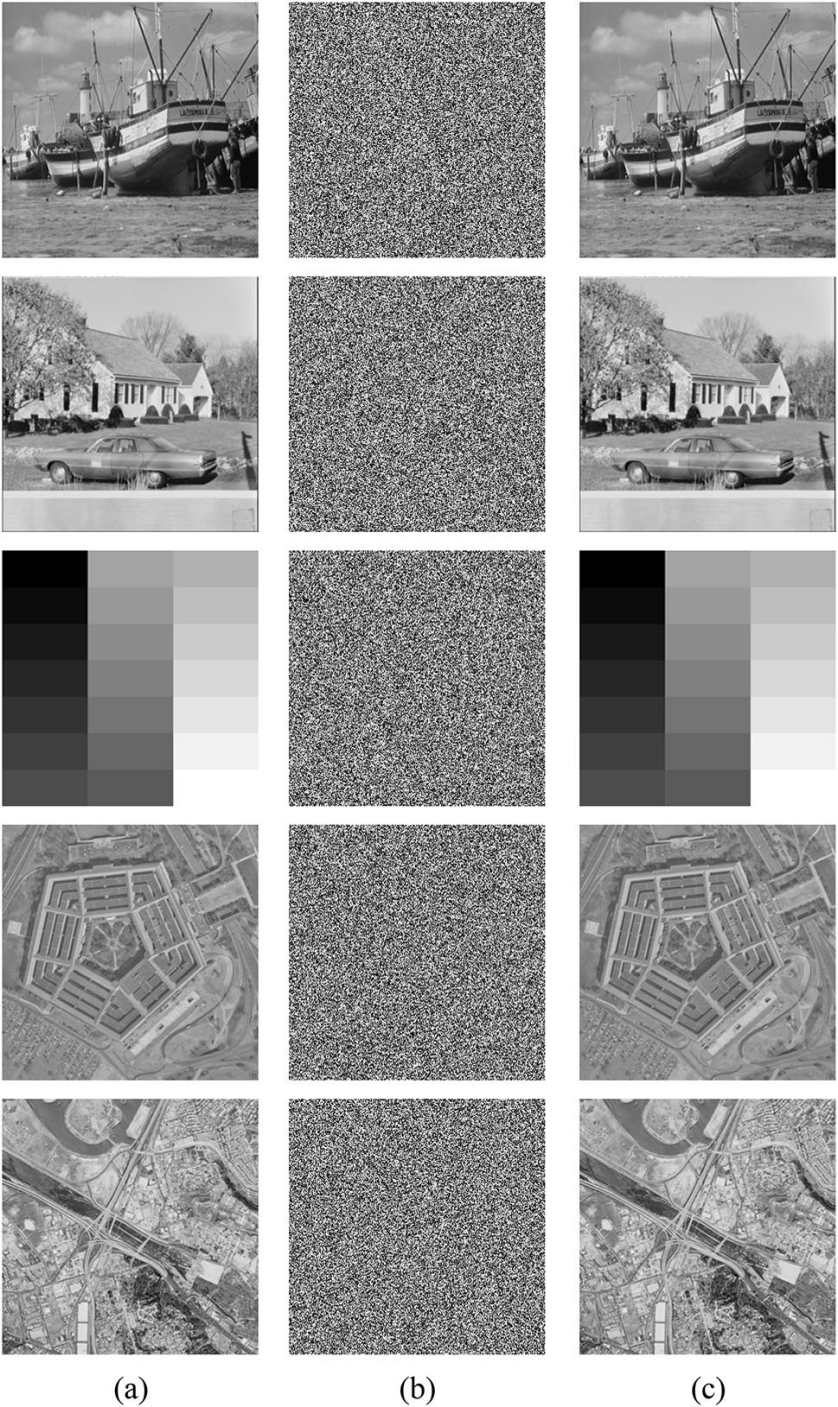
Simulation results: (**a**) plaintext images; (**b**) ciphertext images; (**c**) decrypted images.

To verify the feasibility and effectiveness of this method, we selected five grayscale images (Boat, House, Gray21, Pentagon, SanDiego) with a size of 256 × 256 from the image database (http://sipi.usc.edu/database/). These images were then subjected to encryption validation and tested on the Matlab 2018 platform, with the key given the value of *x*_0_' = *y*_0_' = *z*_0_' = *w*_0_' = 0.01. In terms of security analysis, only the Boat image was used as an example to demonstrate the advantages of our method. The plaintext, ciphertext and decrypted images are shown in Fig. 12, by visual observation, the ciphertext has completely lost the characteristics of the plaintext. The scheme is lossless, and the decrypted image obtained after decrypting the ciphertext is the same as the plaintext.

An ideal encryption scheme must be key-sensitive and resistant to common attacks. Standard performance metrics are used to fully evaluate the proposed encryption scheme, including keyspace analysis, statistical attack analysis, differential attack analysis, and robust analysis.

### Keyspace analysis

The key plays a crucial role in encryption systems and must have a high level of security. The proposed encryption scheme is so sensitive to its key, the plaintext image, and the ciphertext image that any small change in one of them can lead to a significant difference in the generated image. For example, the generated image is random if any slight disturbance is applied to the ciphertext image. The main reason for this is the application of chaotic systems, which are very sensitive to small changes.

An attacker will try to use all possible keys to try to break the encryption system. Therefore, a larger keyspace is more resistant to brute force attacks. It has been shown that even with powerful computers if the keyspace is more significant than 2^128^ ^34^, the encryption method cannot be cracked by a brute force attack within the specified time. In this article, the keystream length reaches 10^60^ > 2^128^, which is sufficient to resist any brute-force attack.

### Statistical analysis

The performance of the proposed image encryption scheme is tested through statistical analysis. Statistical methods to analyze any predictable relationship between plaintext and ciphertext images.

#### Histogram analysis

**Figure 13:**
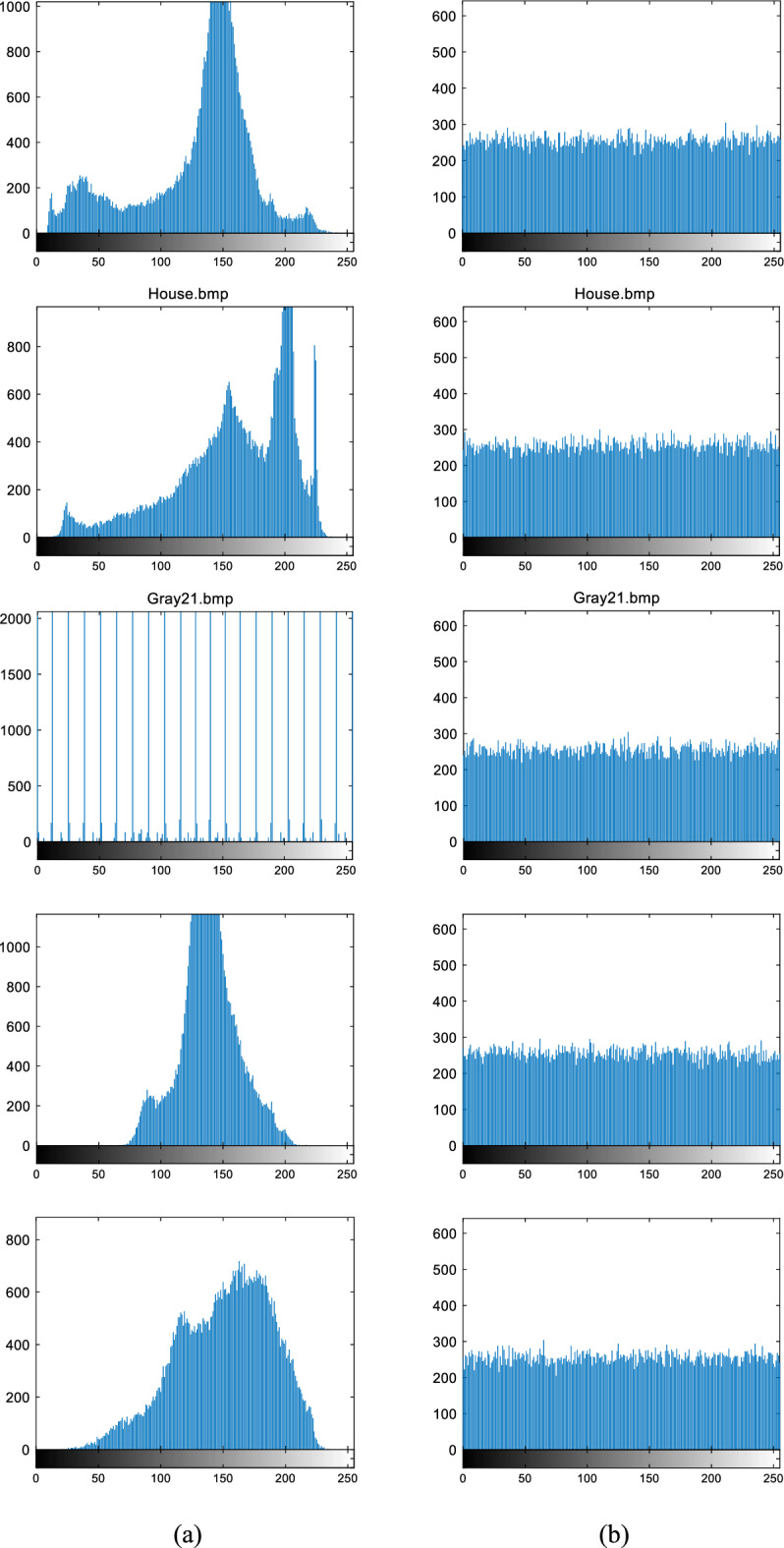
Histogram: (**a**) histogram of plaintext images Boat, House, Gray21, Pentagon, SanDiego; (**b**) histogram of each corresponding ciphertext image.

The histogram represents the distribution of pixels across different grey levels in the image. In a robust cryptosystem, the pixel distribution in the ciphertext image should exhibit uniformity and be distinguishable from the histogram of the plaintext image. It is evident from Fig. 13 that the uniform distribution of pixel values in the ciphertext image prevents attackers from extracting any statistical information.

#### ${{\varvec{\upchi}}}^{2}$test

To prove that this uniformity is not only visually uniform but also theoretically uniformly distributed, the χ^2^ test is performed on the ciphertext. The histogram of the image is represented by *hist*_*i*_(*i* = 0, 1, …, 255). Equation (18) depicts the formula used to calculate the χ^2^ distribution of the histogram.

18 $$\begin{array}{*{20}c} {{\upchi }^{2} = \frac{1}{256}\left( {\mathop \sum \limits_{i = 0}^{255} hist_{i} - \frac{1}{256}\mathop \sum \limits_{i = 0}^{255} hist_{i} } \right)^{2} .} \\ \end{array}$$

The histogram obeys the χ^2^ distribution with 255 degrees of freedom. The hypothesis is accepted given a significance level α such that ${\text{P}}\left\{ {{\upchi }^{2} \ge {\upchi }_{{\upalpha }}^{2} \left( {n - 1} \right)} \right\} = {\upalpha }$, i.e., ${\upchi }^{2} < {\upchi }_{{\upalpha }}^{2} \left( {n - 1} \right)$. When significant level α = 0.01, 0.05 and 0.1, there are ${\upchi }_{0.01}^{2} \left( {255} \right) = 310.45739$, ${\upchi }_{0.05}^{2} \left( {255} \right) = 293.24783$, and ${\upchi }_{0.1}^{2} \left( {255} \right) = 284.33591$.

Displays the χ^2^ distribution of the test images. All ciphertext images in Table 3 pass the test in experiments with significance levels of α = 0.01, α = 0.05, and α = 0.1. The comparison shows that the scheme significantly changes the histogram distribution of the images and can break the statistical features of plaintext images.

**Table 3:** Statistics of χ^2^ distribution of histograms.

	Plaintext images	Ciphertext images	α = 0.01	α = 0.05	α = 0.1
Boat	100,853.4922	243.7891	Passed	Passed	Passed
House	83,975.34375	269.0938	Passed	Passed	Passed
Gray21	614,162.6718	249.7891	Passed	Passed	Passed
Pentagon	151,407.1641	251.3203	Passed	Passed	Passed
SanDiego	59,757.75781	249.3359	Passed	Passed	Passed

#### Correlation analysis

Neighboring pixels of a plaintext image correlate highly in all directions. An ideal encryption scheme aims to minimize the correlation between adjacent pixels in the ciphertext image, thereby effectively enhancing its resistance against statistical attacks. The calculation of the correlation coefficient can be performed using Eq. (19):

19 $$\left\{ {\begin{array}{*{20}l} {E\left( x \right) = \frac{1}{N}\mathop \sum \limits_{i = 1}^{N} x_{i} } \hfill \\ {D\left( x \right) = \frac{1}{N}\mathop \sum \limits_{i = 1}^{N} \left( {x_{i} - E\left( x \right)} \right)^{2} } \hfill \\ {cov\left( {x,y} \right) = \frac{1}{N}\mathop \sum \limits_{i = 1}^{N} \left( {x_{i} - E\left( x \right)} \right)\left( {y_{i} - E\left( y \right)} \right)} \hfill \\ {r_{xy} = \frac{{cov\left( {x,y} \right)}}{{\sqrt {D\left( x \right)D\left( y \right)} }}} \hfill \\ \end{array} } \right.,$$

where *r*_*xy*_ is the correlation coefficient, *cov*(*x*, *y*), *D*(*x*), and *E*(*x*) represent the covariance, variance, and mean value, respectively.

**Figure 14:**
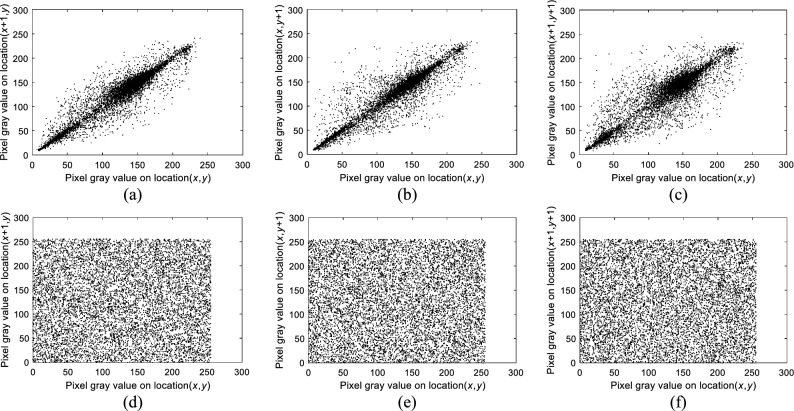
Pixel statistics of randomly selected pixel points and their neighboring pixel points of the Boat image: (**a**–**c**) are the neighboring pixel statistics of the plaintext in horizontal, vertical, and diagonal directions, respectively; (**d**–**f**) are the neighboring pixel statistics of the ciphertext in horizontal, vertical and diagonal directions, respectively.

To analyze adjacent pixel correlation in plaintext and ciphertext images, 10,000 randomly selected adjacent pixel pairs from plaintext and ciphertext images are tested, using the Boat image as an example. Figure 14 demonstrates that neighboring pixels in plaintext images are highly concentrated and have a strong correlation, whereas neighboring pixels in ciphertext images are randomly distributed, resulting in a reduced correlation between them. Furthermore, Table 4 shows that the correlation between neighboring pixels in ciphertext images is lower than in plaintext images.

**Table 4:** Correlation coefficients.

Image	Horizontal	Vertical	Diagonal
Boat			
Plaintext	0.9458	0.9315	0.8861
Ciphertext	0.0043	0.0017	0.0017
House			
Plaintext	0.9359	0.9194	0.8742
Ciphertext	− 0.0085	0.0079	0.0084
Gray21			
Plaintext	0.9998	0.9963	0.9961
Ciphertext	− 0.0052	0.0010	0.0040
Pentagon			
Plaintext	0.8331	0.8089	0.7025
Ciphertext	0.0023	0.0050	− 0.0071
SanDiego			
Plaintext	0.7804	0.7902	0.7071
Ciphertext	0.0039	0.0026	0.0056

#### Information entropy test

Information entropy is a statistical metric used to assess the randomness or disorder of information^35^. It measures the level of uncertainty or unpredictability associated with a given set of information, represented as Eq. (20):

20 $$\begin{array}{*{20}c} {H\left( m \right) = - \mathop \sum \limits_{i}^{l} P\left( {m_{i} } \right)\log_{2} P\left( {m_{i} } \right),} \\ \end{array}$$

where *l* is the grey value of the image, take *l* = 255. *m*_*i*_ is the *i*th grey level value on the image, and *P*(*m*_*i*_) represents the probability of *m*_*i*_.

The ideal information entropy value for a completely random image is 8. By measuring the information entropy of the ciphertext image, we can determine how close it is to 8 and how random the image information is. As shown in Table 5, after encryption, the information entropy of all ciphertext images is close to 8.

**Table 5:** Information entropy.

Image	Information entropy
Boat	
Plaintext	7.1572
Ciphertext	7.9973
House	
Plaintext	7.2298
Ciphertext	7.9970
Gray21	
Plaintext	4.8997
Ciphertext	7.9973
Pentagon	
Plaintext	6.5577
Ciphertext	7.9972
SanDiego	
Plaintext	7.2289
Ciphertext	7.9972

### Differential attack analysis

Differential attack involves studying the impact of differences in the plaintext on its corresponding ciphertext. The objective is to establish a connection between the plaintext and ciphertext images, aiming to identify vulnerabilities and potentially compromise the encryption algorithm. NPCR and UACI are the two methods to test whether the encryption scheme resists differential attacks^36^. NPCR measures the ratio of differing pixels found at corresponding locations in two images, relative to the total number of pixels in the image, with an ideal value of 99.6094%. UACI represents the average density of changes in an image, reflecting the overall intensity of change in the image, with an ideal value of 33.4635%. They are calculated using the Eq. (21):

21 $$\begin{array}{*{20}c} {\left\{ {\begin{array}{*{20}c} {NPCR = \frac{{\mathop \sum \nolimits_{i,j} \left| {P_{1} \left( {i,j} \right) \oplus P_{2} \left( {i,j} \right)} \right|}}{M \times N} \times 100\% } \\ {UACI = \frac{{\mathop \sum \nolimits_{i,j} \left| {P_{1} \left( {i,j} \right) - P_{2} \left( {i,j} \right)} \right|}}{255 \times M \times N} \times 100\% } \\ \end{array} } \right.,} \\ \end{array}$$

where *P*_1_ and *P*_2_ are two different ciphertexts.

Five different ciphertext images of Boat, House, Gray21, Pentagon, and SanDiego are tested, and their NPCR and UACI values for the proposed encryption scheme are presented in Table 6, indicating strong resistance to differential attacks as all test results are close to ideal values.

**Table 6:** NPCR and UACI values.

Scheme	NPCR (%)	UACI (%)
Boat	99.5851	33.4002
House	99.5926	33.3774
Gray21	99.6216	33.5847
Pentagon	99.6033	33.4884
SanDiego	99.6384	33.5156

### Robustness analysis

#### Noise attack analysis

**Figure 15:**
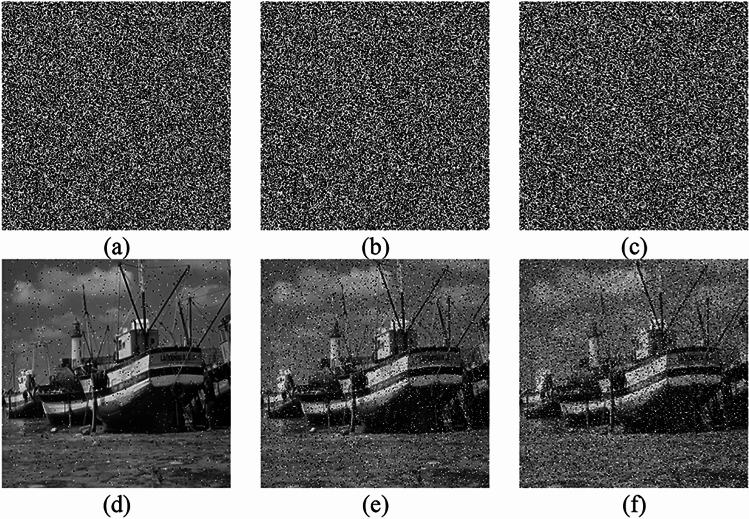
Ciphertext image and decrypted image after pepper noise attack with different strengths: (**a**–**c**) are the ciphertext images with 1%, 5%, and 10% pepper noise added, respectively; (**d**–**f**) are the decrypted images corresponding to (**a**–**c**).

Effective image encryption schemes can reconstruct recognizable decrypted images even when noise interference or data loss occurs during transmission, as digital images may be disrupted by various factors. Pepper noise of 1%, 5%, and 10% are added to the ciphertext image of Boat and then decrypted. Figure 15 shows the experimental results of the images of Boat with noise intensity of 1%, 5%, and 10% and the decrypted image. The figure demonstrates that the decrypted image remains recognizable even under a noise intensity of 10%, indicating the proposed encryption scheme's effective resistance against noise attacks.

#### Clipping attack analysis

**Figure 16:**
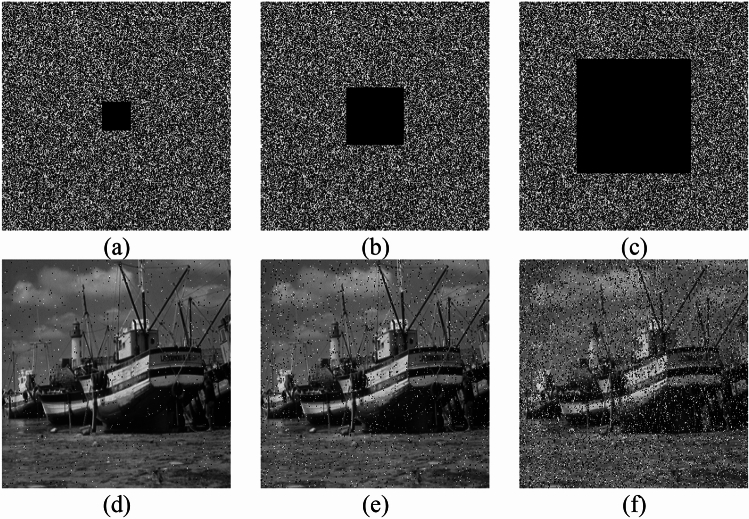
Cropping the ciphertext image and the corresponding decrypted image with different degrees of cropping: (**a**–**c**) are the ciphertext images cropped 1/64, 1/16, and 1/4 respectively; (**d**–**f**) are the decrypted images corresponding to (**a**–**c**).

When images are transmitted over a network, data may be lost for various reasons. By cropping a portion of the ciphertext image and decrypting the cropped ciphertext image, we can test the ability of the ciphertext image to be recovered to the plaintext image in case of data loss and analyze the performance of the encryption scheme against the cropping attack. Cropping attack analysis can reflect the effect of the encryption algorithm on the scrambling of the plaintext image. The better the scrambling effect, the stronger the encryption algorithm recovers the visual features of the plaintext image when a part of the data is lost. In Fig. 16, the ciphertext image of Boat is decrypted after being cropped at different proportions (1/64, 1/16, and 1/4). The decrypted image retains identifiable information, demonstrating the robustness of the proposed encryption scheme against cropping attacks.

### Comparative analysis

We compare the performance of this scheme with the literature of the last 3 years, and the newly compared schemes satisfy the criteria of image security based on both experimental results and performance analysis. They can represent the general level of image encryption security in recent years. Table 7 summarizes the comparative analysis results for the same image, focusing on the evaluation metrics of correlation, NPCR, UACI, and information entropy. It can be seen that, in terms of information entropy, the proposed scheme in this article is higher than others and closer to the ideal value. In terms of NPCR and UACI, the proposed scheme is closer to the ideal value than the others. In terms of relevance, the scheme proposed is better than the schemes^37–40^, but slightly higher than the schemes^41^. Overall, the proposed encryption scheme has a superior performance in terms of security.

**Table 7:** Comparison.

Schemes	Entropy	NPCR	UACI	Correlation coefficient		
				Horizontal	Vertical	Diagonal
Our	7.9974	99.6735	33.2765	0.0018	0.0020	0.0001
^37^	7.9973	99.5859	28.6400	− 0.0035	− 0.0004	− 0.0004
^38^	7.9972	99.6427	33.4741	0.0053	0.0059	0.0031
^39^	7.9974	99.6107	33.4576	− 0.0022	0.0018	− 0.0019
^40^	7.9960	99.5415	33.2400	0.0023	− 0.0020	− 0.0073
^41^	7.9970	99.6277	33.4390	0.0015	− 0.0012	0.0021

## Conclusions

By introducing a new state variable, this article successfully injects a more complex dynamical component into the three-dimensional chaotic system. Through experimental analyses, it is verified that the proposed four-dimensional chaotic system exhibits higher stochasticity and sensitivity. This complexity brings significant benefits to image encryption applications by enhancing the strength and unpredictability of the encryption algorithm. It enables the algorithm to possess a larger keyspace and increases the difficulty of attacks. Furthermore, an image encryption scheme is proposed by combining evolutionary operators with the pseudo-randomness of chaotic systems. In this encryption scheme, the introduction of evolutionary operators effectively disrupts the correlation between neighboring pixels. The resistance to differential attacks is significantly enhanced through operations such as crossover and mutation, yielding remarkable results. The experimental and simulation results comprehensively demonstrate the feasibility and superiority of the scheme. With its vast keyspace and high sensitivity to keys, the scheme effectively withstands multiple attacks, including exhaustive attacks, statistical analysis, and differential attacks.

In summary, the image encryption scheme proposed in this article exhibits commendable performance, meeting the stringent security requirements of image transmission. It is expected to have a positive impact on security measures in practical applications. This research outcome provides valuable insights and practical solutions for further development and innovation in the field of image encryption. Future research could explore additional image security schemes based on chaotic systems and evolutionary strategies to further enhance the security and privacy of image transmission.

## Data Availability

All data generated or analyzed during this study are included in this published article.
